# Clinical Characteristics and Prognostic Analysis of EBV-Positive HIV-Associated Diffuse Large B-Cell Lymphoma in China: A Retrospective Single-Center Study

**DOI:** 10.3390/curroncol33080466

**Published:** 2026-08-05

**Authors:** Lizhi Feng, Haolan He, Han Zhao, Bo Liu, Zhimin Chen, Xinhua Liu, Haisheng Yu, Fengyu Hu, Xiaoping Tang, Linghua Li

**Affiliations:** 1 Infectious Disease Center, Guangzhou Eighth People’s Hospital, Guangzhou Medical University, Guangzhou 510440, China; gz8hflz@126.com (L.F.);; 2 Guangzhou Medical Research Institute of Infectious Diseases, Guangzhou 510440, China; 3 Institute of Infectious Disease, Guangzhou Eighth People’s Hospital, Guangzhou Medical University, Guangzhou 510440, China; 4 Guangzhou Key Laboratory of Clinical Pathogen Research for Infectious Diseases, Guangzhou Eighth People’s Hospital, Guangzhou Medical University, Guangzhou 510440, China

**Keywords:** Epstein–Barr virus, human immunodeficiency virus-associated diffuse large B-cell lymphoma, prognosis, infection, risk stratification

## Abstract

Epstein–Barr virus (EBV) contributes to human immunodeficiency virus (HIV)-associated diffuse large B-cell lymphoma (DLBCL) pathogenesis. We retrospectively analyzed clinical features and outcomes of EBV-positive (*n* = 32) and -negative (*n* = 71) cases. EBV status was determined using in situ hybridization. Immunological parameters, histological subtype, systemic B symptoms, plasma EBV DNA, and response to therapy were examined. Survival was compared using Kaplan–Meier analysis. Factors associated with overall survival (OS) and progression-free survival (PFS) in EBV-positive patients were examined using Cox regression models. EBV-positive cases showed a higher proportion of non-germinal center B cell subtypes and B symptoms, higher circulating EBV viral loads, and lower CD4^+^ T-cell counts at diagnosis than EBV-negative cases. OS did not differ significantly within the full cohort but was shorter in EBV-positive cases with high International Prognostic Index scores or CD4^+^ T-cell counts ≥ 50 cells/μL. Concurrent infections and elevated plasma EBV DNA levels remained associated with unfavorable survival outcomes. EBV-positivity in HIV-associated DLBCL is related to more aggressive clinical and immunological features and independently predicts poorer survival outcomes in high-risk subgroups. Plasma EBV DNA may reliably indicate EBV status and serve as a prognostic biomarker. Integrating these features into clinical decision-making may enhance risk stratification and inform individualized treatments.

## Simple Summary

Diffuse large B-cell lymphoma is an aggressive blood cancer that occurs more often and can be more severe in people living with HIV. Epstein–Barr virus infection may further influence how this disease develops and progresses, but evidence from Chinese patients remains limited. In this retrospective single-center study, we compared Epstein–Barr virus-positive and Epstein–Barr virus-negative HIV-associated diffuse large B-cell lymphoma. We found that EBV-positive patients had weaker immune function, more aggressive clinical features, and higher levels of Epstein–Barr virus DNA in the blood. Although EBV positivity alone did not clearly predict poorer survival in all patients, a higher plasma EBV DNA level was associated with a greater risk of disease progression. These findings suggest that Epstein–Barr virus testing and plasma EBV DNA monitoring may help improve risk assessment, follow-up planning, and future studies on individualized treatment.

## 1. Introduction

Antiretroviral therapy (ART) has reduced the incidence of many human immunodeficiency virus (HIV)-related complications; however, individuals living with HIV still experience elevated rates of aggressive non-Hodgkin lymphoma (NHL), with a 10- to 20-fold higher risk than the general population [1]. Diffuse large B-cell lymphoma (DLBCL) represents the predominant subtype of NHL, constituting 50–70% of HIV-associated NHL cases [2,3]. HIV-associated DLBCL is clinically aggressive, often characterized by rapid progression, biological heterogeneity, and poorer outcomes than DLBCL in immunocompetent individuals [4,5,6].

Epstein–Barr virus (EBV), a widely prevalent γ-herpesvirus with tropism for B lymphocytes, contributes to lymphoma development in immunocompromised individuals. HIV infection impairs immune surveillance, facilitating persistent EBV activation and promoting B-cell infection and transformation [6,7,8]. Approximately 30–50% of HIV-DLBCL cases are associated with EBV infection [9,10,11], compared with approximately 5–15% of DLBCL cases in immunocompetent populations, although the reported prevalence varies geographically [12,13]. EBV is more frequently detected in HIV-associated DLBCL, with EBV-positive cases exhibiting non-germinal center B-cell (GCB) phenotypes, B symptoms, and an elevated tumor burden [9,14]. These findings suggest that EBV contributes to the pathogenesis and progression of HIV-DLBCL in an immunocompromised environment.

Whether EBV affects prognosis in HIV-associated DLBCL remains controversial. Although some reports suggest poorer survival among EBV-positive individuals, others fail to show significant variation. Although several studies from China have described the clinical characteristics and outcomes of HIV-associated DLBCL [15], evidence specifically comparing EBV-defined subgroups in Chinese patients remains limited. In particular, the clinical characteristics and prognostic factors associated with EBV-positive HIV-associated DLBCL have not been well characterized in this population. Against this background, we evaluated the clinical characteristics and prognostic factors of HIV-associated DLBCL according to EBV status in a Chinese cohort. Clarifying the prognostic role of EBV in HIV-associated DLBCL could improve current risk stratification systems and inform individualized therapeutic strategies, thereby contributing to optimized outcomes in this high-risk population.

## 2. Materials and Methods

### 2.1. Study Population

**Figure 1:**
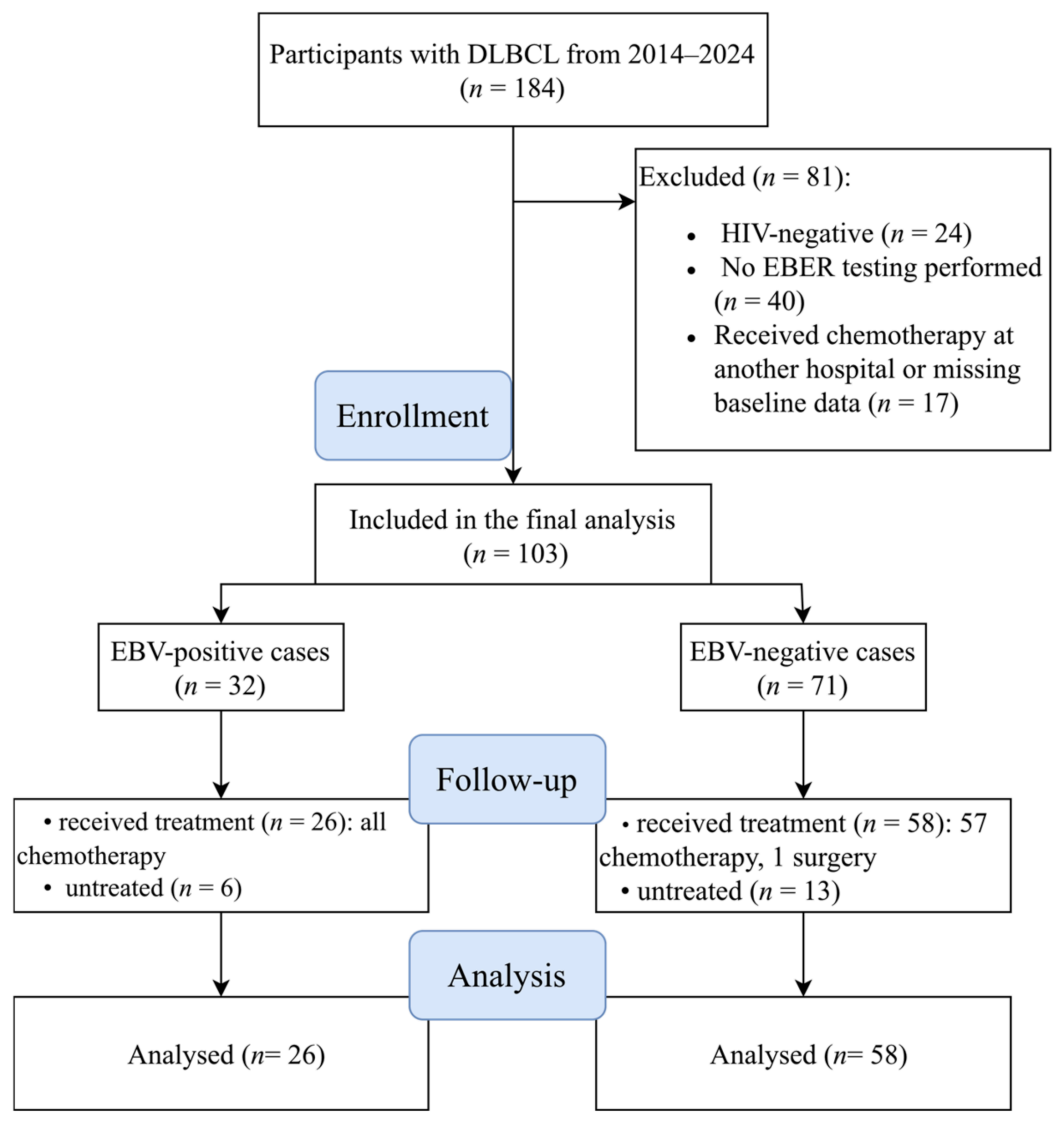
Flow diagram of patient inclusion.

This retrospective study included consecutive patients diagnosed with HIV-DLBCL between January 2014 and December 2024 at the Guangzhou Eighth People’s Hospital of Guangzhou Medical University. During the study period, 184 consecutive patients with pathologically confirmed DLBCL were initially identified at our center; after excluding 24 HIV-negative patients, 160 patients with HIV-associated DLBCL were identified, representing the actual institutional denominator for all patients diagnosed with both HIV infection and DLBCL during the study period. The inclusion criteria were patients aged ≥ 18 years with DLBCL confirmed histologically according to the 2008 or 2016 World Health Organization classification [16,17] who were HIV-positive at the time of diagnosis. Patients were required to have relatively complete clinical, pathological, and laboratory data. Given the retrospective nature of the study, not all individual laboratory parameters were available for every patient; variables with missing data were analyzed based on the number of evaluable cases, and the corresponding denominators were specified in the Results section and tables where applicable. The exclusion criteria included coexisting malignancies, primary immunodeficiency, history of organ transplantation, incomplete baseline information, absence of EBER testing, or receipt of chemotherapy at another hospital before baseline evaluation. Among the 40 patients without EBER results, retrospective testing could not be performed consistently because archived diagnostic tumor tissue was not uniformly available. The patient selection process is summarized in Figure 1.

### 2.2. Pathological and Immunohistochemical Evaluation

Lymphoma specimens preserved in paraffin blocks following formalin fixation were reviewed by senior hematopathologists at our center. To classify tumors into GCB and non-GCB subtypes, immunostaining for CD10, BCL6, and MUM1 was conducted and interpreted using the Hans algorithm. Other markers, including CD20 and Ki-67, were also evaluated.

### 2.3. EBV Status Assessment

EBV status was assessed using in situ hybridization (ISH) for EBV-encoded RNA (EBER) in formalin-fixed paraffin-embedded (FFPE) lymphoma tissue sections. Chromogenic ISH targeting EBER was conducted utilizing a fluorescein-labeled probe kit (catalogue no. ISH-7001, Wuxi OriGene Technologies, Inc., Wuxi, China), following the manufacturer’s instructions. Briefly, FFPE lymphoma tissue sections (4 μm) were deparaffinized, blocked, and subjected to protease digestion. The slides were then hybridized with a fluorescein-labeled EBER probe, and signals were detected. Tumors with positive nuclear staining in ≥20% of tumor cells were defined as EBV-positive (Figure S1) [18,19]. Peripheral blood EBV DNA levels were quantified at the time of DLBCL diagnosis and before the initiation of anti-lymphoma therapy using a commercial real-time fluorescent PCR kit (Sansure Biotech Inc., Changsha, China) according to the manufacturer’s instructions. The assay uses EBV-specific primers and a fluorescent probe targeting a conserved region of the EBV genome. Results were reported as copies/mL. The linear quantitative range of the assay was 4.00 × 10^2^ to 4.00 × 10^9^ copies/mL, and the analytical sensitivity was 4.00 × 10^2^ copies/mL. Plasma EBV DNA levels ≥ 400 copies/mL were considered detectable. Values below the lower limit of quantification were assigned a value of 200 copies/mL, corresponding to one-half of the lower limit of quantification, before log10 transformation for statistical analyses.

### 2.4. Treatment and Follow-Up

All patients underwent ART during and after the lymphoma treatment. Among the 84 patients in the treatment cohort, 29 had already received ART before DLBCL diagnosis, and the remaining 55 initiated ART at or shortly after diagnosis. Because ART exposure was not the primary focus of this study, detailed ART duration, regimen changes, and adherence data were not systematically analyzed. First-line lymphoma regimens primarily include (R)-dose-adjusted (DA)-etoposide, prednisone, vincristine, cyclophosphamide, and hydroxydaunorubicin (EPOCH); DA-EPOCH; R-cyclophosphamide, hydroxydaunorubicin, vincristine, prednisone (CHOP); and CHOP-like protocols tailored to individual patient conditions [20,21]. Follow-up data were obtained from medical records and telephone interviews using the follow-up questionnaire developed for this study (File S1). Overall survival (OS) was defined as the period from initial diagnosis until death from any cause or the most recent follow-up. Progression-free survival (PFS) was defined as the period from initial diagnosis to the earliest event of disease progression, relapse, death, or last clinical evaluation.

### 2.5. Statistical Analysis

Statistical analyses comparing EBV-positive and EBV-negative groups were conducted using Pearson’s chi-square or Fisher’s exact test for categorical variables and the Mann–Whitney U test for continuous variables, while correlations were assessed using Spearman’s rank correlation coefficient.

Survival analyses were conducted using Kaplan–Meier curves and log-rank tests. Univariable analysis was performed to identify potential prognostic factors associated with OS and PFS in the EBV-positive group. Variables with a *p*-value < 0.05 in the Univariable analysis were subsequently included in the multivariable analysis using the Cox proportional hazards regression model. All statistical analyses were performed using SPSS software (version 26.0; IBM Corp., Armonk, NY, USA) and GraphPad Prism (version 10.0; GraphPad Software, San Diego, CA, USA). Statistical significance was set at *p* < 0.05.

## 3. Results

### 3.1. Characteristics of Patients

Among the initially screened cases, 103 patients with HIV-associated DLBCL met the inclusion criteria and were included in the final analysis. The mean (standard deviation) age at diagnosis was 48.5 (12.5) years, and 82.5% were males. Among them, 71.8% were concurrently diagnosed with HIV infection and DLBCL at the time of onset. At the time of diagnosis, the median CD4^+^ T-cell count was 125 cells/mm^3^ (range: 1–931), and 66.0% of assessable individuals had CD4^+^ levels under 200 cells/mm^3^, reflecting a severely immunocompromised state.

Chronic viral coinfections were also observed; hepatitis B surface antigen was detected in 16.5% of cases, and 6.8% of cases had an active hepatitis C virus infection. HIV viral load data were available for 89 patients (86.4%), of whom 30.3% had achieved HIV virological suppression at the time of DLBCL diagnosis, defined as a plasma HIV RNA level < 50 copies/mL.

A total of 32 patients were classified as EBV-positive HIV-associated DLBCL based on positive ISH results for EBER in tumor tissues, whereas the remaining 71 patients were categorized as EBV-negative. In the EBV-positive group, 22 (68.8%) patients had an Eastern Cooperative Oncology Group (ECOG) performance status ≥ 2, 25 (80.6%) had stage III/IV disease (among 31 evaluable patients, one lacked imaging data for staging), 13 (41.9%) had two or more extranodal sites (among 31 evaluable patients), and 24 (75.0%) had elevated lactate dehydrogenase (LDH) levels (among 32 patients). Additionally, 10 (31.3%) patients had bone marrow involvement, 4 (12.5%) had central nervous system (CNS)/leptomeningeal involvement, 24 (75.0%) presented with B symptoms, and 15 (51.7%) were classified as non-GCB type (among 29 patients with available data).

In the EBV-negative group, 45 (63.4%) patients had an ECOG performance status ≥ 2, 50 (71.4%) had stage III/IV disease (among 70 evaluable patients, one lacked imaging data for staging), 30 (42.9%) had two or more extranodal sites (among 70 evaluable patients), and 49 (70.0%) had elevated LDH levels (among 70 evaluable patients). Seventeen (23.9%) patients had bone marrow involvement, four (5.6%) had CNS/leptomeningeal involvement, 29 (40.8%) presented with B symptoms, and 18 (25.4%) were classified as non-GCB.

Owing to incomplete clinical data, one patient in each group lacked imaging information, resulting in 31 evaluable EBV-positive cases and 70 EBV-negative cases for staging, extranodal involvement, and LDH assessment. Additionally, only 29 EBV-positive and 71 EBV-negative cases had available cell-of-origin classification data. The missing data did not completely overlap across the variables.

EBV-positive patients exhibited significantly lower CD4^+^ T-cell counts and Th/Ts ratios, more frequent non-GCB phenotypes, and a higher incidence of B symptoms than EBV-negative patients (*p* < 0.05). Furthermore, elevated plasma EBV DNA was more common among EBV-positive cases than among EBV-negative cases (*p* < 0.0001). Consistent with this finding, among patients with available plasma EBV DNA measurements, plasma EBV DNA levels were significantly higher in the EBER-positive group than in the EBER-negative group (median, 3.91 [IQR, 2.77–5.16] vs. 2.30 [IQR, 2.30–2.30] log10 copies/mL; *p* < 0.001) (Figure S2). Plasma EBV DNA levels were positively correlated with tumor EBER status (Spearman’s ρ = 0.747, *p* < 0.001). Table 1 presents the comparison of clinical characteristics between the groups.

### 3.2. Survival Analysis

A total of 84 patients with HIV-associated DLBCL who received treatment were included in the survival analysis, comprising 26 EBV-positive and 58 EBV-negative cases. The remaining 19 patients, including 6 EBV-positive and 13 EBV-negative patients, did not receive anti-lymphoma therapy, primarily because of poor performance status, advanced disease at presentation, or financial constraints. Among them, 83 patients (80.6%) received systemic chemotherapy, while one patient underwent surgical resection for a presumed gastric carcinoma. Postoperative pathology confirmed DLBCL, but the patient declined subsequent chemotherapy. Comparison between the EBV-positive and EBV-negative subgroups revealed no significant differences in the use of chemotherapy regimens, immunotherapy (rituximab), ART, or CNS prophylaxis (*p* > 0.05; Table S1). At a median follow-up of 22.5 months (range: 0.1–119.7 months), 53 patients (51.5%) died. The estimated 2-year overall OS and PFS rates for the cohort were 43.0% and 35.5%, respectively.

**Figure 2:**
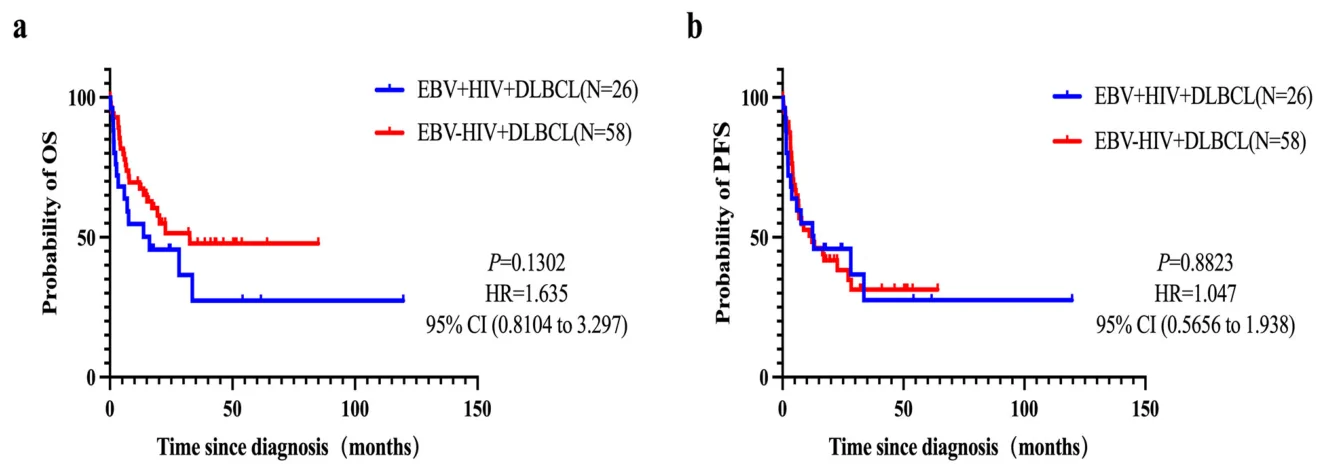
Kaplan–Meier survival curves in EBV-positive and EBV-negative HIV-associated DLBCL patients. (**a**) Overall survival (OS) and (**b**) progression-free survival (PFS) in EBV-positive (blue) and EBV-negative (red) HIV-associated diffuse large B-cell lymphoma (DLBCL) patients who underwent anti-lymphoma therapy. Survival time was calculated from the initiation of anti-lymphoma therapy.

**Figure 3:**
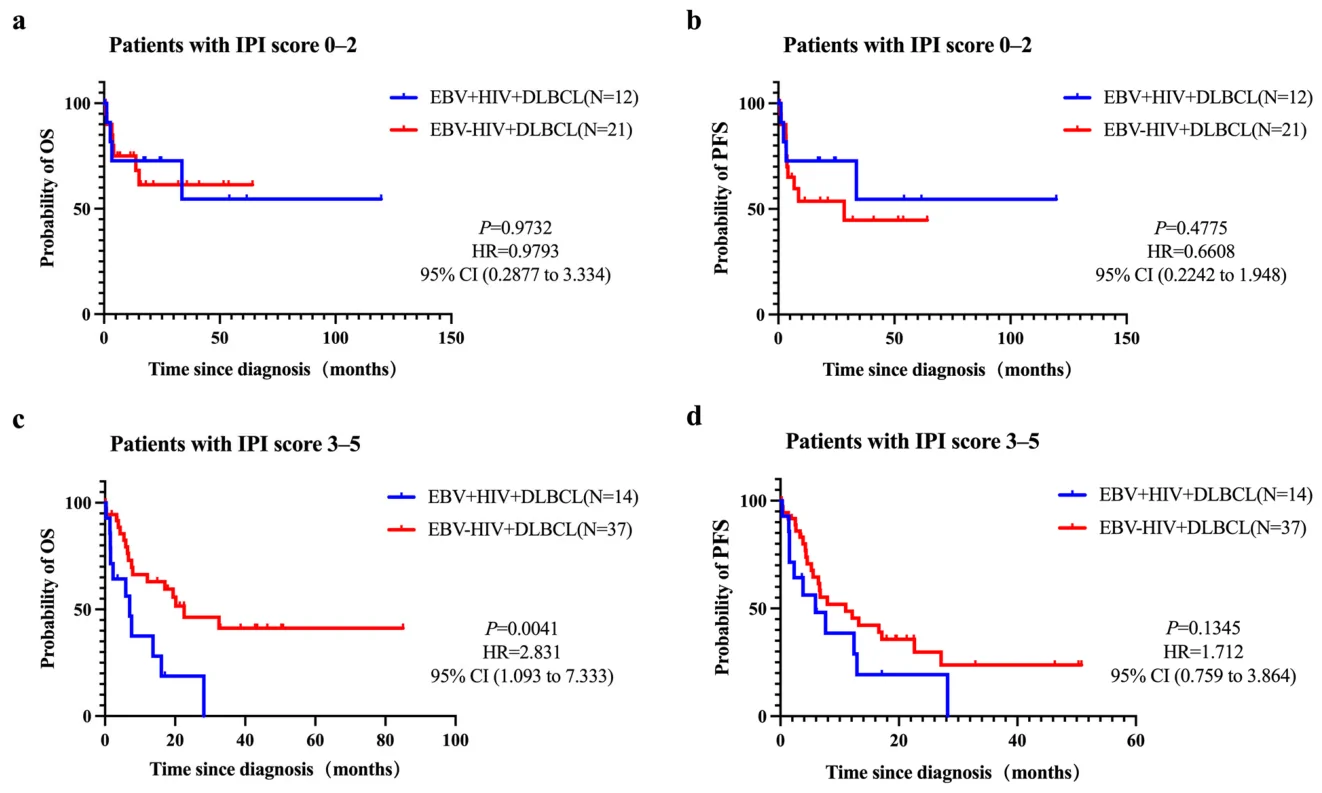
Kaplan–Meier curves comparing survival outcomes between EBV-positive and EBV-negative HIV-associated DLBCL patients who underwent anti-lymphoma therapy, stratified by IPI risk group. (**a**) OS in the low-risk group, defined as IPI score 0–2; (**b**) PFS in the low-risk group; (**c**) OS in the high-risk group, defined as IPI score 3–5; (**d**) PFS in the high-risk group.

**Figure 4:**
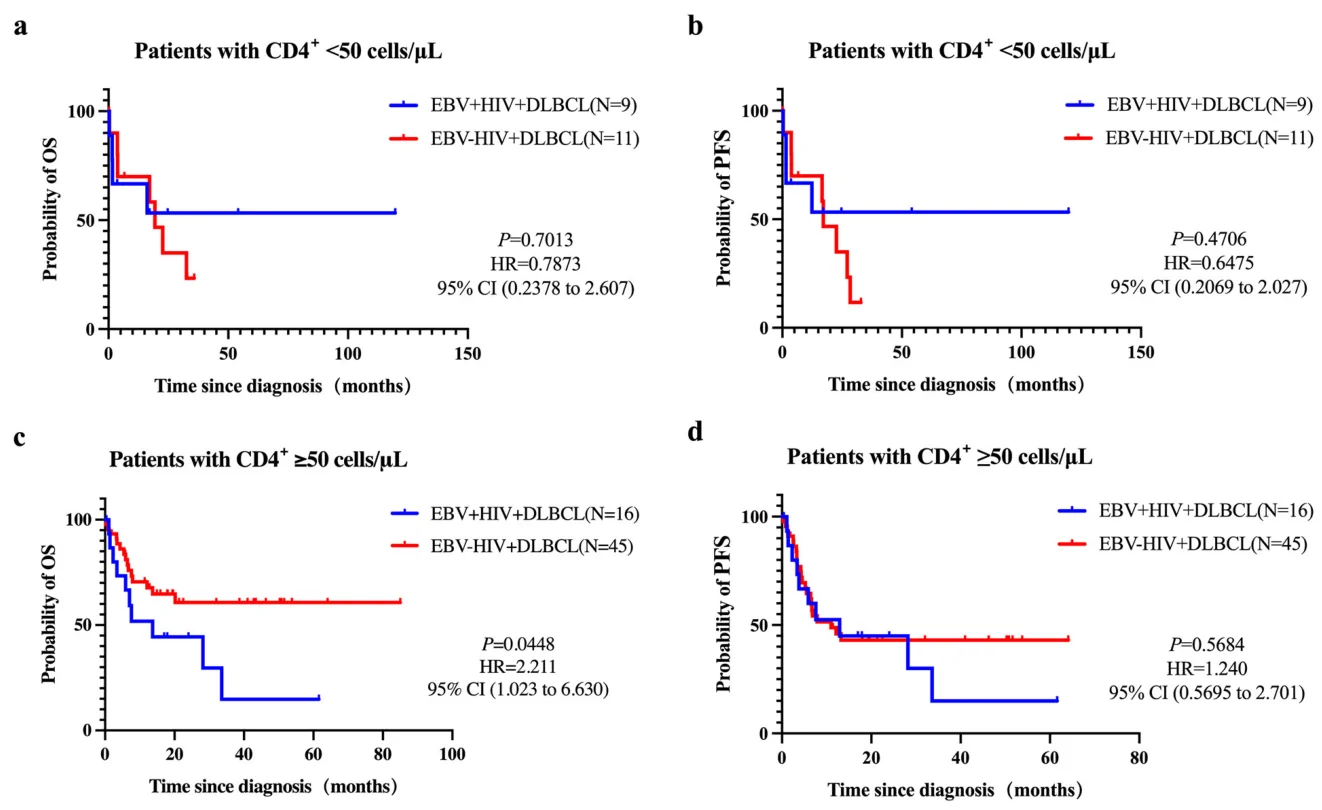
Kaplan–Meier curves comparing survival outcomes between EBV-positive and EBV-negative HIV-associated DLBCL patients who underwent anti-lymphoma therapy, stratified by CD4^+^ T-cell count. (**a**) OS in patients with CD4^+^ T-cell count < 50 cells/μL; (**b**) PFS in patients with CD4^+^ T-cell count < 50 cells/μL; (**c**) OS in patients with CD4^+^ T-cell count ≥ 50 cells/μL; (**d**) PFS in patients with CD4^+^ T-cell count ≥ 50 cells/μL.

No significant variation in OS or PFS was observed when comparing EBV-positive and EBV-negative individuals (median OS: 16.1 vs. 32.5 months, *p* = 0.1302; median PFS: 12.9 vs. 12.1 months, *p* = 0.8823) (Figure 2a,b). Stratified analysis by the International Prognostic Index (IPI) score revealed that in the high-risk group (IPI score, 3–5), EBV-positive patients demonstrated lower median OS than EBV-negative patients (7 vs. 22.6 months; *p* = 0.0041), whereas PFS was not significantly different between the groups (*p* = 0.2874). In the low-risk group (IPI 0–2), no significant differences in OS or PFS were observed between the EBV-positive and EBV-negative subgroups (Figure 3a–d). When stratified by immune status, among patients with CD4^+^ T-cell counts ≥ 50 cells/mm^3^, EBV-positive patients showed a significantly shorter OS than EBV-negative patients (median OS: 13.7 months vs. not reached, *p* = 0.0448), with no significant difference in PFS (*p* = 0.2521). Among patients with CD4^+^ T cell < 50 cells/mm^3^, OS and PFS did not differ significantly between the EBV-positive and EBV-negative groups (Figure 4a–d). By contrast, when stratified by the Ann Arbor stage (I–II vs. III–IV), no significant differences in OS or PFS were observed between EBV-positive and EBV-negative patients in either subgroup (Figure S3a–d). In an additional sensitivity analysis restricted to patients who had received ART before DLBCL diagnosis and achieved HIV virologic suppression at diagnosis (plasma HIV RNA < 50 copies/mL), no significant differences in OS or PFS were observed between the EBV-positive and EBV-negative groups (Figure S4a,b).

Given the limited sample size and number of outcome events, exploratory multivariable Cox regression analyses were performed to identify factors associated with OS and PFS among the 26 treated patients with EBV-positive HIV-associated DLBCL. Variables found to be significant in the univariable analysis (*p* < 0.05) were included in the model, including high-risk IPI score, ECOG performance status (≥2 vs. <2), elevated LDH and lactate levels, concurrent infections, elevated plasma EBV DNA levels, hypoalbuminemia, and lack of CNS prophylaxis.

In the multivariable model for OS, concurrent infections at DLBCL diagnosis remained associated with poorer OS (hazard ratio [HR], 5.689; 95% confidence interval [CI]: 1.027–31.502, *p* = 0.047), and elevated plasma EBV DNA levels also remained associated with poorer OS (HR, 2.701; 95% CI: 1.286–5.670, *p* = 0.009) (Table 2). By contrast, in the multivariable model for PFS, only elevated plasma EBV DNA levels remained associated with poorer PFS (HR, 2.688; 95% CI: 1.269–5.695, *p* = 0.01) (Table 3).

## 4. Discussion

In this study, we comprehensively characterized the clinical features, treatment strategies, and prognostic factors of HIV-associated DLBCL, with a focus on EBV status, over an 11-year period at a single academic medical center. Although previous studies have suggested that EBV promotes lymphomagenesis in the context of HIV through impaired immune surveillance, chronic activation, and persistent B-cell infection [22,23], its precise prognostic value in HIV-associated DLBCL remains uncertain. Our findings demonstrate that patients with DLBCL and HIV frequently exhibit advanced clinical features at diagnosis, including impaired functional status, the presence of systemic B symptoms, elevated serum LDH, later Ann Arbor stages, and higher IPI classifications. These findings are consistent with those of previous studies reporting aggressive clinical behavior in HIV-associated lymphomas [4,24]. When stratified by EBV status, patients with EBV-positive HIV-associated DLBCL showed a significantly higher incidence of B symptoms than those with EBV-negative DLBCL.

Previous Chinese studies have largely focused on the clinical characteristics and outcomes of HIV-associated DLBCL as a whole, while direct comparisons according to EBV status remain limited [12]. In our cohort, EBV status was not significantly associated with OS or PFS overall, whereas higher plasma EBV DNA levels were independently associated with poorer survival within the EBV-positive subgroup. These findings provide additional clinical and prognostic data on EBV-positive HIV-associated DLBCL in Chinese patients.

In a French cohort, the majority (79%) of individuals with HIV-DLBCL had already commenced ART by the time of lymphoma diagnosis, with a median CD4^+^ count of 233 cells/mm^3^ [25]. By contrast, in our study, only 28.2% of patients were already on ART at the time of diagnosis, with lower baseline levels of both CD4^+^ T-cell counts and CD4^+^/CD8^+^ (Th/Ts) ratios—mean values were 125 cells/mm^3^ and 0.22, respectively. This disparity may partly reflect delayed HIV diagnosis and ART initiation in our cohort, with some patients being diagnosed only after the development of lymphoma or opportunistic infections. EBV-positive HIV-associated DLBCL cases in our cohort presented with significantly lower CD4^+^ T-cell counts (median 73 vs. 144 cells/mm^3^, *p* = 0.033) and more severely reduced Th/Ts ratios at diagnosis (median 0.16 vs. 0.26, *p* = 0.029), reflecting greater immune impairment in the EBV-positive group. These findings are consistent with previous reports of lower CD4+ T-cell counts in EBV-positive HIV-associated DLBCL [14]. However, effective ART and virological suppression do not fully eliminate the risk of HIV-associated lymphoma, and residual immune dysfunction may remain relevant to EBV-associated lymphomagenesis [26,27]. Additionally, in our cohort, patients with EBV-positive HIV-associated DLBCL demonstrated a significantly higher incidence of the non-GCB subtype than EBV-negative cases, which aligns with a previous study reporting that approximately 70% of EBV-positive HIV-DLBCL cases are non-GCB with frequent STAT3 mutations, whereas EBV-negative cases tend to be of GCB origin [14]. Moreover, pre-ART and pre-rituximab era data from Lindsay et al. [9] also demonstrated a close association between EBV positivity and the activated B-cell/non-GCB phenotype. Experimental studies have shown that EBV infection can activate JAK/STAT and NF-κB signaling in DLBCL cells. Because activation of these pathways is also characteristic of activated B-cell/non-GCB DLBCL, this may partly explain the association between EBV positivity and the non-GCB phenotype [28]. The higher frequency of B symptoms in EBV-positive patients may be partly related to cytokine dysregulation. Previous studies have shown increased IL-10 expression in EBV-positive DLBCL tumors, while higher serum IL-10 levels have been observed in DLBCL patients with B symptoms [29,30].

EBV infection is detected in 30–50% of HIV-associated DLBCL cases [9,10,11], compared with approximately 10% in HIV-negative DLBCL populations [31], underscoring the stronger association between EBV and DLBCL in the setting of HIV infection. In our cohort, 37.5% of patients with HIV-associated DLBCL had elevated plasma EBV DNA levels. Detectable plasma EBV DNA was more frequent in the EBV-positive group than in the EBV-negative group (90.9% vs. 17.2%, *p* < 0.001). Consistent with this, plasma EBV DNA levels were significantly higher in patients with EBER-positive tumors and were positively correlated with tumor EBER status. These findings support a close association between circulating EBV burden and tumor EBER positivity in HIV-associated DLBCL. Similar findings have been reported in previous studies [32,33,34], supporting the utility of plasma EBV DNA as a biomarker for clinical monitoring and disease assessment.

Beyond its association with tumor EBV status, elevated plasma EBV DNA has also been linked to poorer survival in HIV-associated lymphomas [35,36,37]. One study [35] reported that an EBV load > 5000 copies/mL at lymphoma diagnosis independently predicted shorter OS and PFS. Consistent with these findings, our exploratory multivariable Cox analyses showed that higher plasma EBV DNA levels remained associated with poorer OS and PFS among treated patients with EBV-positive HIV-associated DLBCL. Additionally, Kaplan–Meier survival curve analyses were performed to elucidate the prognostic implications of EBV status. Although OS did not differ between EBV-positive and EBV-negative individuals in the entire cohort, stratified analysis showed that EBV-positive status was associated with shorter OS among those with high IPI scores (3–5) or CD4^+^ T-cell counts ≥ 50 cells/mm^3^. These findings suggest that the prognostic relevance of EBV may vary across clinical subgroups defined by IPI score and immune status. Because a high IPI score reflects the combined effects of several adverse clinical features, the unfavorable prognostic influence associated with EBV may be more apparent in patients who already have a high-risk clinical profile, whereas no significant differences in OS or PFS were observed in the low-risk group. Among patients with CD4^+^ T-cell counts ≥ 50 cells/mm^3^, the shorter OS observed in EBV-positive cases may also be related to the adverse biological and immunological features associated with EBV-positive disease, including greater immune impairment, a higher frequency of the non-GCB subtype, and higher plasma EBV DNA levels. By contrast, among patients with CD4^+^ T-cell counts < 50 cells/mm^3^, severe immunodeficiency and infection-related complications may have exerted a greater influence on survival in both groups, reducing the apparent difference associated with EBV status. The difference in OS without a corresponding difference in PFS may reflect the influence of factors beyond lymphoma progression. After disease progression, survival may also be affected by treatment tolerance and complications related to HIV-associated immune dysfunction. Concurrent infections, which were associated with worse OS in the EBV-positive group, may have further contributed to the observed difference in OS. In addition, the relatively small sample sizes after stratification may have limited the ability to detect differences in PFS.

The CD4^+^ T-cell threshold of 50 cells/mm^3^ was selected because profound immunosuppression below this level is associated with a high risk of infection-related complications and may influence lymphoma treatment, including the use of rituximab [20,21,38]. Stratification by this threshold allowed us to explore whether the prognostic association of EBV differed according to the degree of immune suppression. Additionally, therapeutic approaches, comprising chemotherapy, rituximab administration, ART, and CNS prophylaxis, did not differ significantly between the EBV-positive and EBV-negative groups (*p* > 0.05 for all). These findings suggest that the subgroup survival differences observed are unlikely to be explained solely by differences in treatment approaches and may also reflect differences in disease biology and host-related factors.

In this context, our previous study [39] demonstrated that EBV infection is associated with upregulation of immune checkpoint molecules, including PD-1, PD-L1, and PD-L2, which correlated with worse OS in acquired immunodeficiency syndrome-associated NHL. These findings suggest that EBV may contribute to tumor immune escape through immune checkpoint pathways. This observation provides a biological rationale for further investigation of immune checkpoint blockade, including anti-PD-1 therapy, in EBV-positive HIV-associated DLBCL, particularly in high-risk patients, although its clinical efficacy in this setting remains to be established.

The survival outcomes in our cohort were less favorable than those reported in several contemporary studies of HIV-associated DLBCL. In the CALL-001 cohort, the 2-year PFS and OS rates were 46.8% and 58.0%, respectively, whereas other studies in the modern ART era have reported higher survival rates [15,40]. In comparison, the estimated 2-year PFS and OS rates in our cohort were 35.5% and 43.0%, respectively. These relatively unfavorable outcomes may reflect the real-world characteristics of our study population, including advanced-stage disease, extranodal involvement, impaired immune status, concurrent infections, and other high-risk clinical features. The unfavorable outcomes observed among EBV-negative patients should not be interpreted as an adverse effect of EBV negativity itself. These patients still had HIV-associated DLBCL and remained exposed to adverse prognostic factors related to lymphoma burden and HIV-associated immune dysfunction. Thus, EBV-negative status does not define a clinically low-risk population in this setting.

HIV-specific immunosuppressive factors, including concurrent infections, have emerged as important prognostic factors for HIV-associated lymphomas [41]. In line with previous findings, such as those by Dendle et al. [42], who demonstrated that infections independently predict mortality in patients with DLBCL regardless of HIV status, our study found that concurrent infections were independently associated with worse OS in EBV-positive HIV-associated DLBCL. For PFS, the association did not reach statistical significance (HR, 5.534; 95% CI, 0.993–30.843; *p* = 0.051), possibly reflecting the limited size of the EBV-positive subgroup.

Our study has some limitations. Its retrospective, single-center design inherently introduced potential selection and information biases. The multivariable analyses were based on only 26 treated patients, and the limited number of outcome events may have increased the risk of model overfitting and imprecise effect estimates. Therefore, these findings should be considered exploratory and require validation in larger multicenter cohorts. EBV status was determined by EBER ISH and plasma EBV DNA quantification; however, but no functional studies were performed to elucidate the molecular mechanisms linking EBV infection to lymphomagenesis and immune dysfunction. Treatment regimens and follow-up durations were not entirely uniform. In addition, 19 patients did not receive anti-lymphoma therapy for various clinical or patient-related reasons, and the restriction of survival analyses to treated patients may have introduced selection bias.

## 5. Conclusions

This study on HIV-associated DLBCL in China underscores the clinical and prognostic significance of EBV positivity. EBV-positive patients showed deeper immunosuppression, more severe B symptoms, and a predominance of the non-GCB subtype. Elevated plasma EBV DNA levels correlated strongly with tumor EBV status and remained associated with poorer OS and PFS. Despite standardized treatment across groups, EBV positivity was associated with worse outcomes in the high-risk subgroups. These findings highlight the need for tailored management strategies for patients with EBV-positive HIV-associated DLBCL.

## Figures and Tables

**Table 1:** Baseline clinicopathological features of EBV-positive and EBV-negative HIV-associated DLBCL patients.

Characteristic	Total (*n* = 103)	EBV+HIV-DLBCL (*n* = 32)	EBV-HIV-DLBCL (*n* = 71)	t/U/χ^2^	*p*
Demographics					
Male, *n* (%)	85 (82.5)	29 (90.6)	56 (78.9)	2.112	0.146
Age at diagnosis in years, mean (SD)	48.47 (12.51)	46.53 (12.63)	49.34 (12.45)	−1.054	0.294
HIV factors					
CD4 count, median (IQR)	125 (48.25–243.5)	73 (17–161)	144 (54–246.5)	784	0.033
Th/Ts, median (IQR)	0.22 (0.103–0.378)	0.16 (0.06–0.32)	0.26 (0.14–0.405)	777.5	0.029
HIV Virological suppression ^a^, *n*/*N* (%)	27/89 (30.3)	9/28 (34.6)	18/61 (29.5)	0.222	0.637
Pre-diagnosis ART, *n* (%)	29 (28.2)	8 (25)	21 (29.6)	0.228	0.633
Concurrent hepatitis B, *n* (%)	17 (16.5)	8 (25)	9 (12.7)	2.431	0.119
Concurrent hepatitis C, *n* (%)	7 (6.8)	1 (3.1)	6 (8.5)	—	0.431
Lymphoma factors					
Lymphoma subtype, *n*/*N* (%) ^b^				6.477	0.011
GCB type	67/100 (67)	14/29 (48.3)	53/71 (74.6)		
Non-GCB type	33/100 (33)	15/29 (51.7)	18/71 (25.4)		
ECOG PS ≥ 2, *n* (%)	67 (65)	22 (68.8)	45 (63.4)	0.280	0.597
Stage, *n*/*N* (%) ^c^				0.955	0.329
1–2	26/101 (25.7)	6/31 (19.4)	20/70 (28.6)		
3–4	75/101 (74.3)	25/31 (80.6)	50/70 (71.4)		
Number of extranodal sites, *n*/*N* (%) ^c^				0.007	0.931
0–1	58/101 (57.4)	18/31 (58.1)	40/70 (57.1)		
2 or more	43/101 (42.6)	13/31 (41.9)	30/70 (42.9)		
IPI, *n* (%)				0.103	0.748
Not high risk	41 (39.8)	12 (37.5)	29 (40.8)		
High risk	62 (60.2)	20 (62.5)	42 (59.2)		
CNS/leptomeningeal involvement, *n* (%)	8 (7.8)	4 (12.5)	4 (5.6)	—	0.251
Bone marrow involvement, *n* (%)	27 (26.2)	10 (31.3)	17 (23.9)	0.609	0.435
B symptoms, *n* (%)	53 (51.5)	24 (75)	29 (40.8)	10.30	0.001
LDH (U/L), median (IQR)	442 (233.5–910.5)	378 (240.8–951.5)	485 (232.8–895)	1117	0.983
Plasma EBV DNA					
Available, *n*	80	22	58		
Detectable plasma EBV DNA ^d^, *n* (%)	30 (37.5)	20 (90.9)	10 (17.2)	—	<0.0001

EBV, Epstein–Barr virus; DLBCL, diffuse large B-cell lymphoma; HIV, human immunodeficiency virus; IQR, interquartile range; GCB, germinal center B-cell-like; ECOG PS, Eastern Cooperative Oncology Group Performance Status; IPI, International Prognostic Index; CNS, central nervous system; LDH, lactate dehydrogenase; VL, viral load. Continuous variables are presented as median (IQR). Categorical variables are presented as *n* (%) unless otherwise specified. For variables with missing data, percentages were calculated using the number of evaluable patients as the denominator. ^a^ HIV viral load data were available for 89 patients. HIV virological suppression was defined as a plasma HIV RNA level < 50 copies/mL at the time of DLBCL diagnosis. ^b^ Cell-of-origin classification data were available for 29 patients in the EBV-positive group and 71 patients in the EBV-negative group. ^c^ Ann Arbor stage and the number of extranodal sites were evaluable in 31 patients in the EBV-positive group and 70 patients in the EBV-negative group; one patient in each group lacked imaging data required for staging and extranodal assessment. ^d^ Plasma EBV DNA measurements were available for 80 patients. Detectable plasma EBV DNA was defined as a level ≥ 400 copies/mL at the time of DLBCL diagnosis.

**Table 2:** Univariable and multivariable analyses of factors affecting overall survival (OS) in EBV-positive HIV-associated DLBCL patients (*n* = 26).

Variable	Univariate Analysis			Multivariate Analysis		
	HR	95% CI	*p*	HR	95% CI	*p*
Age	0.7564	0.307–1.864	0.5131			
High Risk IPI	4.852	1.376–17.105	0.014			
ECOG PS	2.285	1.312–3.979	0.004			
Number of extranodal sites ≥ 2	0.982	0.399–2.417	0.968			
B symptoms	1.933	0.629–5.938	0.25			
Bone marrow involvement	0.901	0.358–2.267	0.825			
CNS involvement	1.448	0.418–5.017	0.559			
Stage III or IV	1.703	0.468–6.204	0.419			
Non-GCB (Hans)	0.657	0.271–1.590	0.351			
cART before lymphoma	0.407	0.135–1.23	0.111			
Rituximab use	0.542	0.220–1.333	0.182			
CNS prophylaxis	0.201	0.066–0.615	0.005			
Concurrent infections ^a^	2.572	1.015–6.521	0.046	5.689	1.027–31.502	0.047
CD4 count	0.998	0.995–1.002	0.296			
HIV Virological suppression	0.384	0.122–1.209	0.102			
Elevated LDH	1.001	1–1.001	0.002			
LAC	1.405	1.156–1.709	0.001			
EBV-VL	3.034	1.460–6.303	0.003	2.701	1.286–5.670	0.009
HGB	0.990	0.971–1.008	0.275			
ALB	0.846	0.770–0.930	0.001			

OS, overall survival; EBV, Epstein–Barr virus; DLBCL, diffuse large B-cell lymphoma; HR, hazard ratio; CI, confidence interval; IPI, International Prognostic Index; ECOG PS, Eastern Cooperative Oncology Group Performance Status; CNS, central nervous system; LDH, lactate dehydrogenase; LAC, lactic acid; VL, viral load; HGB, hemoglobin; ALB, albumin. ^a^ Concurrent infections were defined as active infections present at the time of DLBCL diagnosis and included bacterial infections, tuberculosis, nontuberculous mycobacterial infections, *Pneumocystis jirovecii* pneumonia, other fungal infections, and cytomegalovirus infection.

**Table 3:** Univariable and multivariable analyses of factors affecting progression-free survival (PFS) in EBV-positive HIV-associated DLBCL patients (*n* = 26).

Variable	Univariate Analysis			Multivariate Analysis		
	HR	95% CI	*p*	HR	95% CI	*p*
Age	1.009	0.971–1.048	0.648			
High Risk IPI	4.629	1.317–16.27	0.017			
ECOG PS	2.236	1.297–3.852	0.004			
Number of extranodal sites ≥ 2	0.941	0.382–2.314	0.894			
B symptoms	1.969	0.64–6.059	0.238			
Bone marrow involvement	0.862	0.343–2.169	0.753			
CNS involvement	1.431	0.413–4.957	0.571			
Stage III or IV	1.677	0.460–6.114	0.433			
Non-GCB (Hans)	0.657	0.271–1.592	0.352			
cART before lymphoma	0.402	0.133–1.218	0.107			
Rituximab use	0.526	0.214–1.297	0.163			
CNS prophylaxis	0.198	0.065–0.609	0.005			
Concurrent infections ^a^	2.586	1.018–6.568	0.046	5.534	0.993–30.843	0.051
CD4 count	0.998	0.995–1.002	0.285			
HIV Virological suppression	0.380	0.12–1.199	0.099			
Elevated LDH	1.001	1–1.001	0.002			
LAC	1.415	1.159–1.727	0.001			
EBV-VL	3.015	1.445–6.288	0.003	2.688	1.269–5.695	0.01
HGB	0.99	0.972–1.009	0.309			
ALB	0.848	0.772–0.932	0.001			

OS, overall survival; EBV, Epstein–Barr virus; DLBCL, diffuse large B-cell lymphoma; HR, hazard ratio; CI, confidence interval; IPI, International Prognostic Index; ECOG PS, Eastern Cooperative Oncology Group Performance Status; CNS, central nervous system; LDH, lactate dehydrogenase; LAC, lactic acid; VL, viral load; HGB, hemoglobin; ALB, albumin. ^a^ Concurrent infections were defined as active infections present at the time of DLBCL diagnosis and included bacterial infections, tuberculosis, nontuberculous mycobacterial infections, *Pneumocystis jirovecii* pneumonia, other fungal infections, and *cytomegalovirus* infection.

## Data Availability

The data that support the findings of this study are not publicly available due to privacy and ethical restrictions. Deidentified data may be made available from the corresponding author upon reasonable request and with approval from the Ethics Committee of Guangzhou Eighth People’s Hospital, Guangzhou Medical University.

## Supplementary Materials

The following supporting information can be downloaded at: https://www.mdpi.com/article/10.3390/curroncol33080466/s1, Figure S1: EBER in situ hybridization confirming EBV infection in HIV-associated DLBCL. Representative Epstein–Barr virus-encoded RNA (EBER) in situ hybridization showing strong nuclear positivity in ≥20% of lymphoma cells, consistent with EBV-positive disease. Original magnification, ×400; Figure S2: Comparison of plasma EBV DNA levels between EBER-positive and EBER-negative HIV-associated DLBCL. Plasma EBV DNA levels were compared between patients with EBER-positive and EBER-negative tumors. Each dot represents an individual patient. Horizontal lines indicate the median, with error bars representing the interquartile range (IQR). Differences between groups were assessed using the Mann–Whitney U test; Figure S3: Kaplan–Meier curves comparing survival outcomes by EBV status in HIV-associated DLBCL patients stratified by Ann Arbor stage. Kaplan–Meier curves showing overall survival (OS) and progression-free survival (PFS) in EBV-positive and EBV-negative patients who underwent anti-lymphoma therapy, stratified by Ann Arbor stage. (a) OS in patients with early-stage disease (stage I–II); (b) PFS in patients with early-stage disease; (c) OS in patients with advanced-stage disease (stage III–IV); (d) PFS in patients with advanced-stage disease; Figure S4: Kaplan–Meier curves comparing survival outcomes by EBV status in HIV-associated DLBCL patients with prior ART and virologic suppression. Kaplan–Meier curves showing overall survival (OS) and progression-free survival (PFS) in EBV-positive and EBV-negative patients who underwent anti-lymphoma therapy, had received antiretroviral therapy (ART) before DLBCL diagnosis, and achieved virologic suppression (HIV viral load <50 copies/mL) at diagnosis. (a) OS; (b) PFS; File S1: Follow-up Questionnaire for Patients with HIV-associated DLBCL (Brief Version); Table S1: Treatment characteristics of EBV-positive and EBV-negative HIV-associated DLBCL.

## Abbreviations

The following abbreviations are used in this manuscript:

ART	Antiretroviral therapy
CNS	Central nervous system
DLBCL	Diffuse large B-cell lymphoma
EBER	EBV-encoded RNA
EBV	Epstein–Barr virus
ECOG	Eastern Cooperative Oncology Group
FFPE	Formalin-fixed paraffin-embedded
GCB	Germinal center B
HIV	Human immunodeficiency virus
HR	Hazard ratio
IPI	International Prognostic Index
ISH	In situ hybridization
LDH	Lactate dehydrogenase
NHL	Non-Hodgkin lymphoma
OS	Overall survival
PS	Progression-free survival
