# Supplementary material for: Clinical Characteristics and Prognostic Analysis of EBV-Positive HIV-Associated Diffuse Large B-Cell Lymphoma in China: A Retrospective Single-Center Study
Source: Curr Oncol. 2026 Aug 5;33(8):466. doi: 10.3390/curroncol33080466 (PMC13510999; doi:10.3390/curroncol33080466)
Supplement: Supplementary file 1 [file curroncol-33-00466-s001.zip › Supplementary Figure S1-S4.pdf]

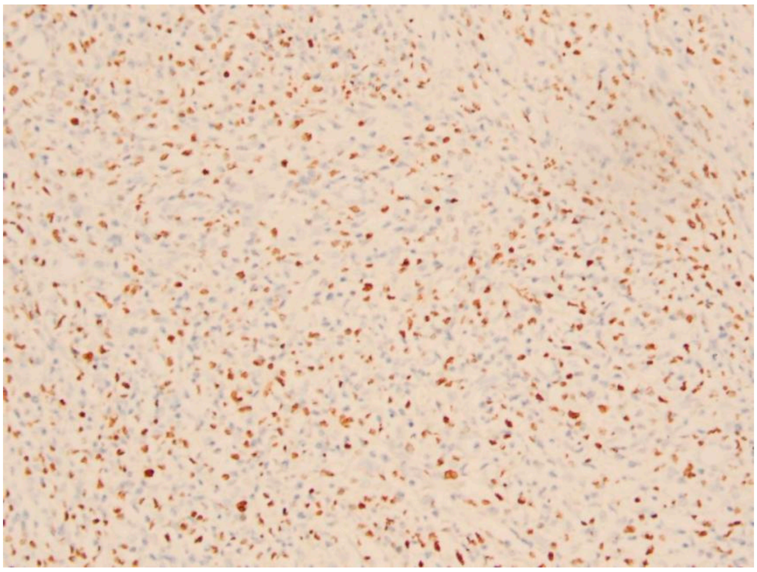

***Supplementary Figure S1.* EBER in situ hybridization confirming EBV infection in HIV-associated DLBCL.** Representative Epstein–Barr virus-encoded RNA (EBER) in situ hybridization showing strong nuclear positivity in ≥20% of lymphoma cells, consistent with EBV-positive disease. Original magnification, ×400.

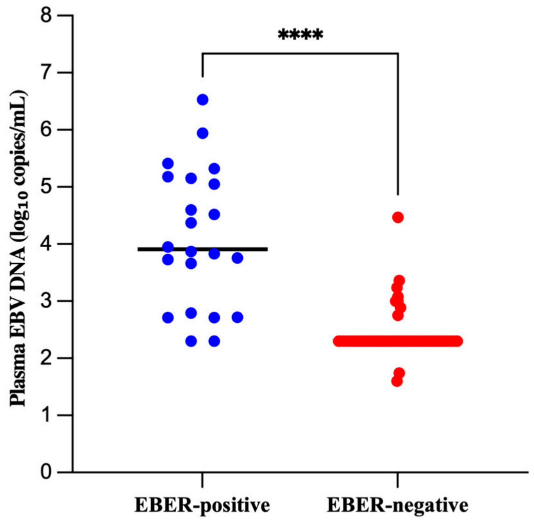

Supplementary Figure S2. **Comparison of plasma EBV DNA levels between EBER-positive and EBER-negative HIV-associated DLBCL.** Plasma EBV DNA levels were compared between patients with EBER-positive and EBER-negative tumors. Each dot represents an individual patient. Horizontal lines indicate the median, with error bars representing the interquartile range (IQR). Differences between groups were assessed using the Mann–Whitney U test.

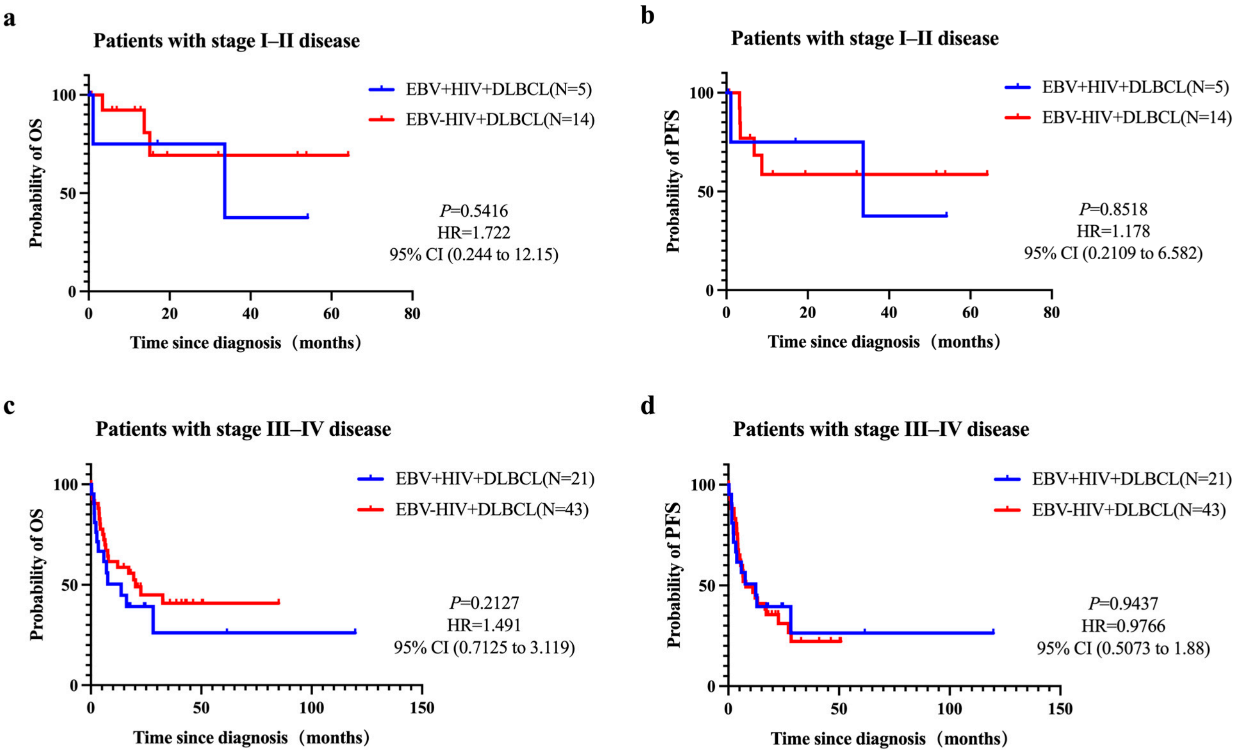

Supplementary Figure S3. **Kaplan–Meier curves comparing survival outcomes by EBV status in HIV-associated DLBCL patients stratified by Ann Arbor stage.** Kaplan–Meier curves showing overall survival (OS) and progression-free survival (PFS) in EBV-positive and EBV-negative patients who underwent anti-lymphoma therapy, stratified by Ann Arbor stage. (a) OS in patients with early-stage disease (stage I–II); (b) PFS in patients with early-stage disease; (c) OS in patients with advanced-stage disease (stage III–IV); (d) PFS in patients with advanced-stage disease.

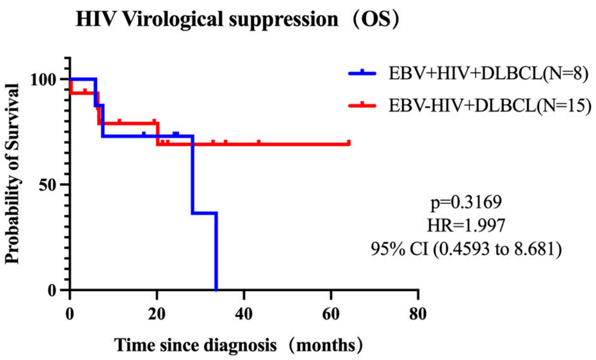

(a)

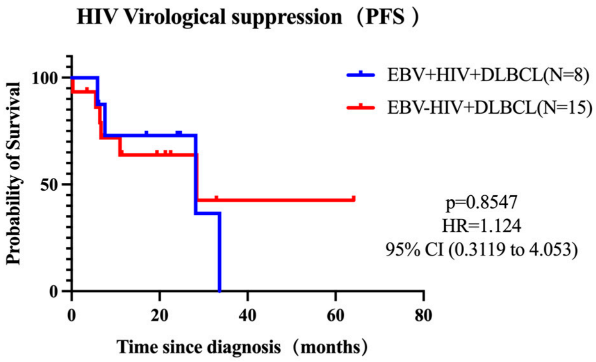

(b)

Supplementary Figure S4. **Kaplan–Meier curves comparing survival outcomes by EBV status in HIV-associated DLBCL patients with prior ART and virologic suppression.** Kaplan–Meier curves showing overall survival (OS) and progression-free survival (PFS) in EBV-positive and EBV-negative patients who underwent anti-lymphoma therapy, had received antiretroviral therapy (ART) before DLBCL diagnosis, and achieved virologic suppression (HIV viral load <50 copies/mL) at diagnosis. (a) OS; (b) PFS.
