# Supplementary material for: Clinical Characteristics and Prognostic Analysis of EBV-Positive HIV-Associated Diffuse Large B-Cell Lymphoma in China: A Retrospective Single-Center Study
Source: Curr Oncol. 2026 Aug 5;33(8):466. doi: 10.3390/curroncol33080466 (PMC13510999; doi:10.3390/curroncol33080466)
Supplement: Supplementary file 1 [file curroncol-33-00466-s001.zip › Supplementary File S1.Follow-up Questionnaire.pdf]

Follow-up Questionnaire for Patients with HIV-associated DLBCL (Brief Version)

Patient ID: __________
Follow-up Date: __________
Mode of Follow-up: Phone__________

I、Patient Survival Status

1. Current survival status：

· ☐ Alive → to be completed only for patients alive

· ☐ Deceased → only complete death information

2. If Deceased, please complete

· Date of death：__________

· Cause of death：__________

II、Quality of Life

1. Current overall health status (self-assessed):

☐ Good

☐ Fair

☐ Poor

III、Disease Status (to be completed only for patients alive)

1. Last follow-up date: _______

· Place of follow-up：

☐ In our hospital ☐ Outside hospital

2. Has there been lymphoma relapse or progression since the last follow-up?

☐ Yes ☐ No

· If Yes，please specify：

Date of relapse/progression：__________

Symptoms/signs of relapse：__________

Site of relapse：__________

3. Any new B symptoms (fever, night sweats, >10% weight loss)?

☐ Yes ☐ No

IV、 Treatment Status(to be completed only for patients alive)

1. ART status：

☐ On ART ☐ Not on ART

2. Regular monitoring of HIV viral load and CD4+ T-cell count?

☐ Yes

☐ No

· Latest CD4 count：__________

3. Was the last anti-lymphoma treatment performed in our hospital?

☐ Yes

☐ No，If No, please specify the treatment location：__________

4. Date & regimen of last anti-lymphoma treatment: __________

Interviewer Name: __________
