# Supplementary material for: Clinical Characteristics and Prognostic Analysis of EBV-Positive HIV-Associated Diffuse Large B-Cell Lymphoma in China: A Retrospective Single-Center Study
Source: Curr Oncol. 2026 Aug 5;33(8):466. doi: 10.3390/curroncol33080466 (PMC13510999; doi:10.3390/curroncol33080466)
Supplement: Supplementary file 1 [file curroncol-33-00466-s001.zip › Supplementary Table S1.pdf]

Supplementary Table S1. Treatment characteristics of EBV-positive and EBV-negative HIV-associated DLBCL

Treatment Approach	Total (n = 103)	EBV+ HIV-DLBCL(n = 32)	EBV− HIV-DLBCL (n = 71)	χ^2	P
Chemotherapy , n (%)	83（80.6）	26（81.3）	57（80.3）	0.013	0.908
Cycles of chemotherapy, n (%)				2.41	0.3
1–3 cycles	27 (32.)	10(38.5)	17(29.8)		
4–6 cycles	17 (20.)	7 (26.9)	10 (17.5)		
>6 cycles	39 (47)	9 (34.6)	30(52.6)		
CHOP / R-CHOP regimen, n (%)	45 (43.7)	12(37.5)	33 (46.5)	0.723	0.395
DA-EPOCH ± R regimen, n (%)	35 (34)	7 (21.9)	28 (39.4)	3.032	0.082
Rituximab use, n (%)	69（67）	20（62.5）	49（69）	0.423	0.515
Receive ART, n (%)	88（85.4）	28（87.5）	60（84.5）	—	0.772
CNS prophylaxis via intrathecal injection, n (%)	44（42.7）	12（37.5）	32（45.1）	0.517	0.472

* EBV, Epstein–Barr virus; DLBCL, diffuse large B-cell lymphoma; HIV, human immunodeficiency virus; R, Rituximab; CHOP, cyclophosphamide, doxorubicin, vincristine and prednisolone; CNS, central nervous system; DA-EPOCH, etoposide, prednisolone, vincristine, cyclophosphamide, doxorubicin; ART, antiretroviral therapy; CNS, central nervous system.
